# Perforation of the ascending colon during implantation of an indwelling peritoneal catheter: a case report

**DOI:** 10.1186/s12876-020-01489-4

**Published:** 2020-10-16

**Authors:** Maria Paparoupa, Henning Wege, Anna Creutzfeldt, Marcial Sebode, Faik G. Uzunoglu, Olaf Boenisch, Axel Nierhaus, Jakob R. Izbicki, Stefan Kluge

**Affiliations:** 1 Department of Intensive Care Medicine, University Medical Center Hamburg-Eppendorf, Martinistr. 52, 20246 Hamburg, Germany; 2 Department of Internal Medicine, Gastroenterology, Infectiology and Tropical Diseases, University Medical Center Hamburg-Eppendorf, Martinistr. 52, 20246 Hamburg, Germany; 3 Department of General, Visceral and Thoracic Surgery, University Medical Center Hamburg-Eppendorf, Martinistr. 52, 20246 Hamburg, Germany

**Keywords:** Refractory ascites, Liver cirrhosis, Paracentesis, Ascites drainage, Tunneled catheter

## Abstract

**Background:**

Tunneled peritoneal drainage catheters are described as an effective and relatively safe method in the management of malignant and non-malignant refractory ascites. Therapeutic advantages, linked to their use, are self-management of ascites and palliative care at home. Complications occur rarely. We describe an ascending colon perforation after implantation of a peritoneal drainage in a patient with refractory ascites due to liver cirrhosis.

**Case presentation:**

The 68-year-old male was admitted to the intensive care unit due to severe community acquired pneumonia. The ascites drainage was inserted in order to reduce the intra-abdominal pressure and enable appropriate ventilation. A few hours later, bowel content could be detected in the tube and an abdominal computed tomography confirmed the intestinal perforation. Notably, there was no pneumoperitoneum and peritonitis had not yet set in. The catheter was removed during an emergency laparotomy and sutured closure of both perforation sites was performed.

**Conclusion:**

Patients with septated ascites and intraperitoneal adhesions are at potential higher risk of bowel perforation during implantation of an indwelling peritoneal catheter. A mini-laparotomy is, therefore, necessary in order to ensure safe implantation and positioning of the catheter in those cases.

## Background

Tunneled peritoneal drainage catheters are described as effective and relatively safe in the management of malignant and non-malignant refractory ascites [1]. It has been shown that the need for paracentesis in patients with refractory ascites, due to terminal liver disease, may be reduced without compromising renal function [2]. In the setting of malignant ascites, tunneled peritoneal drainage catheters facilitate self-management and palliative care at home [3]. The most common complications been reported are catheter-associated infections, fluid leakage around the entry side, dislodgement or accidental loss, occlusion or sheathing of the catheter, and groin pain [1, 3]. We describe the rare complication of an ascending colon perforation during implantation of a tunneled peritoneal drainage catheter in a patient with refractory ascites due to cirrhosis of the liver.

## Case presentation

The 68-year-old male was admitted to the intensive care unit with severe community acquired pneumonia. Because of rapidly progressing respiratory failure he was intubated and mechanically ventilated. Cryptogenic liver cirrhosis had been diagnosed several years ago and paracentesis had to be conducted repeatedly, due to refractory ascites, in the preceding months. In order to enable an appropriate lung-protective ventilation, we decided to establish drainage of the massive ascites, by placing a tunneled peritoneal catheter, as a measure to reduce the intra-abdominal pressure of the patient. Because of septated ascites only small amounts of fluid could be removed and thus a permanently-tunneled catheter was placed (ASEPT® Peritoneal Drainage System, 15.5 F 5.2 mm × 71 cm, pfm medical mepro Gmbh, Am Söterberg 4, 66620 Nonnweiler, Germany). The procedure was completed according to insertion instructions under ultrasonographic guidance.

**Fig. 1:**
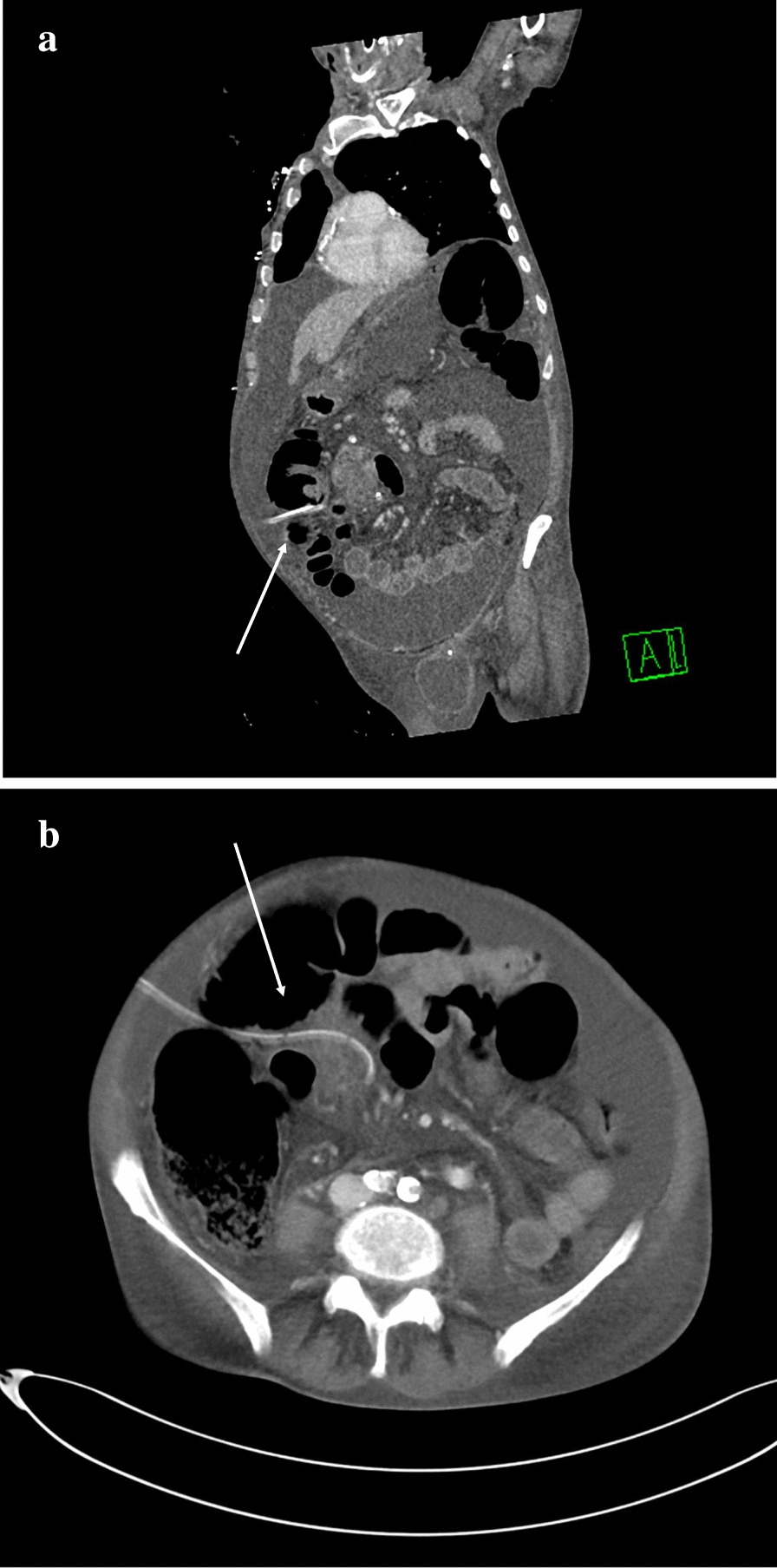
**a** Coronal computed tomography, showing the entry side of the catheter into the abdominal cavity, next to ascending colon (arrow). **b** The catheter (arrow) passing through the bowel wall and inserting peritoneal cavity again

Initially, clear ascites was evacuated. However, a few hours later no more peritoneal fluid could be removed even after flushing the tube with sterile normotonic saline. Instead, bowel content was detected, arousing the suspicion of an intestinal perforation. An abdominal computed tomography confirmed perforation of the ascending colon and showed that the catheter had been passed through the bowel wall and re-entered the peritoneal cavity (Fig. 1a, b). Pneumoperitoneum was absent and peritonitis had not yet set in. Laboratory parameters of the fluid obtained through the catheter were: total cell count < 200 cells/mm^3^, polymorphonuclear cell count < 100 cells/mm^3^, Lipase 50 IU/l, Amylase 24 IU/l.

The patient underwent an emergency laparotomy, where removal of the catheter and sutured closure of both perforation sites was performed. A segmental colectomy was not required, as an extended bowel injury could be intraoperatively excluded at this point. On the first postoperative day, the patient received a transjugular intrahepatic portosystemic shunt (TIPS), in order to facilitate a long-lasting reduction of his portal hypertension. However, a re-laparotomy with right hemicolectomy had to be conducted on the second post-operative day, as an insufficiency of the previously sutured perforation sites occurred. The patient finally died after two months in intensive care, as an irreversible malnutrition state resulted to a chronic respiratory failure.

## Discussion and conclusion

We describe an early major complication during the insertion of a percutaneous tunneled peritoneal catheter, which is otherwise a safe method to manage malignant and non-malignant refractory ascites in patients without known adhesions [1–3]. A case series of two patients with pneumoperitoneum, following an ascites drainage implantation, has been reported before [4]. In both cases pneumoperitoneum developed without bowel perforation and disappeared under conservative management. A possible mechanism could have been the air tracking along the subcutaneous course of the drainage.

Notably, no peritonitis or pneumoperitoneum occurred in our case, as both insertion sites may have been impacted by the catheter itself. Clear ascites was initially drained, since the catheter tip was located in peritoneum and the elapsed time until removal was obviously too short to enable the development of peritonitis.

A similar case of a 57-year-old male with refractory ascites due to advanced liver cancer has been reported before [5]. The patient experienced an iatrogenic colonic perforation after peritoneal drainage catheter insertion, which was successfully treated with endoscopic clipping. The approach of the insertion sites in this case was performed under combined laparoscopic and endoscopic guidance. Although an explorative laparotomy is often considered as the last resort in patients with Child–Pugh C liver cirrhosis, it was the treatment of choice in our case, in order to avoid an extended contamination of the peritoneum with concomitant severe sepsis and to radically remove septated ascites, aiming to the improvement of the critical respiratory situation.

In retrospect, we suppose that our patient was at higher risk of perforation, as the chronically septated ascites had induced some fibrinous-adherence of the bowel to the abdominal wall and subileus was present at the time point of the transcutaneous puncture. We suggest that patients with septated ascites and intraperitoneal adhesions are at higher risk of bowel perforation during implantation of indwelling peritoneal catheters. A mini-laparotomy should be, therefore, utilized in those patients in order to ensure safe implantation and correct positioning of the catheter.

## Data Availability

All data analyzed during this study are included in this manuscript.

## Abbreviation

TIPS: Transjugular intrahepatic portosystemic shunt
